# A closer look at chemotherapy‐induced flagellate dermatitis

**DOI:** 10.1002/ski2.92

**Published:** 2022-01-19

**Authors:** A. Constantinou, D. Kotecha, P. Laouris, B. de Paula

**Affiliations:** ^1^ School of Clinical Medicine University of Cambridge Cambridge UK; ^2^ Department of Oncology University of Cambridge Cambridge UK

## Abstract

**Background:**

Flagellate dermatitis (FD) is a rare skin rash, which may occur following the administration of antineoplastic agents. It has been reported following the administration of bleomycin, docetaxel, trastuzumab, cisplatin, bendamustine and doxorubicin. We provide a summary of the epidemiology, aetiology, pathophysiology, and distribution of chemotherapy‐induced FD.

**Methods:**

PubMed was searched using ((flagellat*) AND (Dermat*)) OR ((Flagellat*) AND (Erythema)). The search yielded 206 publications, out of which 54 individual case reports were identified which fulfilled our inclusion criteria. Statistical analysis was performed where appropriate.

**Results:**

Female patients were slightly more likely to develop FD compared to males. In the majority of cases FD appeared on the upper and lower limbs and pruritus was an accompanying feature in 51% of cases. Most cases developed after the first cycle of chemotherapy and females were statistically more likely to present within the first 72 hr (*p* <0.05). Skin biopsies were taken in 41% of cases and this was not statistically associated with the patient’s gender, (*p* = 0.651), presentation within 72 hr (*p* = 0.076) or cancer diagnosis. Chemotherapy was stopped in 62% of patients and was associated with female gender (*p* = 0.0098). Most patients who received treatment were managed with topical steroids. Time for rash resolution ranged from a few weeks to four months following the discontinuation of the causative drug.

**Conclusion:**

FD is a rare adverse skin effect of chemotherapeutic treatment, most commonly presenting on the upper and lower limbs of patients following their first cycle of chemotherapy. Early presentation is more common in females leading to increased likelihood of stopping chemotherapy. Biopsy findings poorly correlate with disease severity. Continuation of chemotherapy treatment in combination with topical steroids may not adversely affect rash resolution.

## Graphical abstract

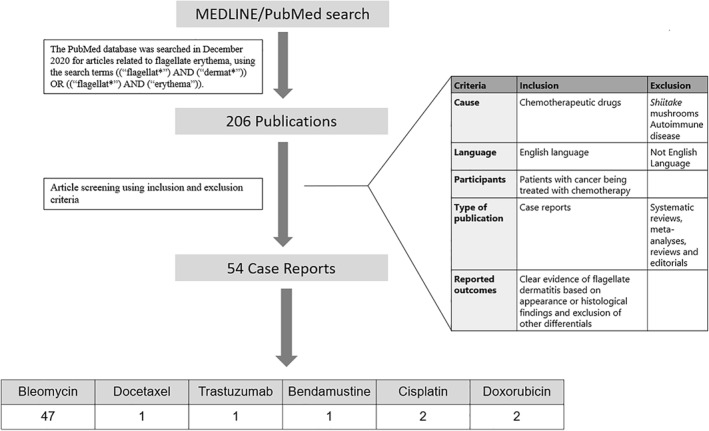

1

**What is already known about this topic?**

- Flagellate dermatitis is a rare skin rash, consisting of pruritic linear hyperpigmentations of the skin in a flagellate configuration.
- Chemotherapy‐induced flagellate dermatitis predominantly occurs following the administration of bleomycin, but has also been reported in association with docetaxel, doxorubicin, trastuzumab, bendamustine and cisplatin.
- Possible mechanisms for this include localised skin accumulation and toxicity.
- Current therapeutic options include cessation of the causative agent, topical or systemic corticosteroids or anti‐histamines.

## 2 INTRODUCTION

Flagellate dermatitis (FD) is a rare skin rash, which can occur following the administration of antineoplastic agents. It is estimated that FD occurs in 1%–2% of oncology patients.^1^ Bleomycin has historically been associated with FD, and was first described in 1970.^2^ The reported incidence in bleomycin‐treated patients lies between 8% to 22%,^1^ but this has been decreasing recently perhaps parallelling the declining use of bleomycin.^3^ In addition to bleomycin, FD has been reported following administration of doxorubicin,^4^, ^5^ trastuzumab,^6^ docetaxel,^7^ cisplatin^8^ and bendamustine.^9^ Additional aetiologies of FD are summarized in Table 1.

**TABLE 1:** Summary of differential aetiology of flagellate dermatitis (modified from Bhushan et al.^10^)

Causes of FD	Examples
Pruritic dermatoses	Dermatographism, phytodermatitis, poison ivy
Toxin‐induced	*Shiitake* mushrooms, Portugese man‐of‐war, jellyfish
Rheumatological conditions	Dermatomyositis, adult‐onset Still's disease
Viral infections	HIV
Other	Chikungunya fever, idiopathic

FD is described as a pruritic linear hyperpigmentation of flagellate pattern. It has a characteristic ‘whip‐like’ appearance formed by rows of firm papules.^3^ As the erythema subsides, deep pigmentation may occur and persist for up to 6 months.^11^, ^12^

Here, we review 54 cases of chemotherapy‐associated FD to evaluate the spectrum of reported clinical manifestations and management.

## 3 MATERIALS AND METHODS

**FIGURE 1:**
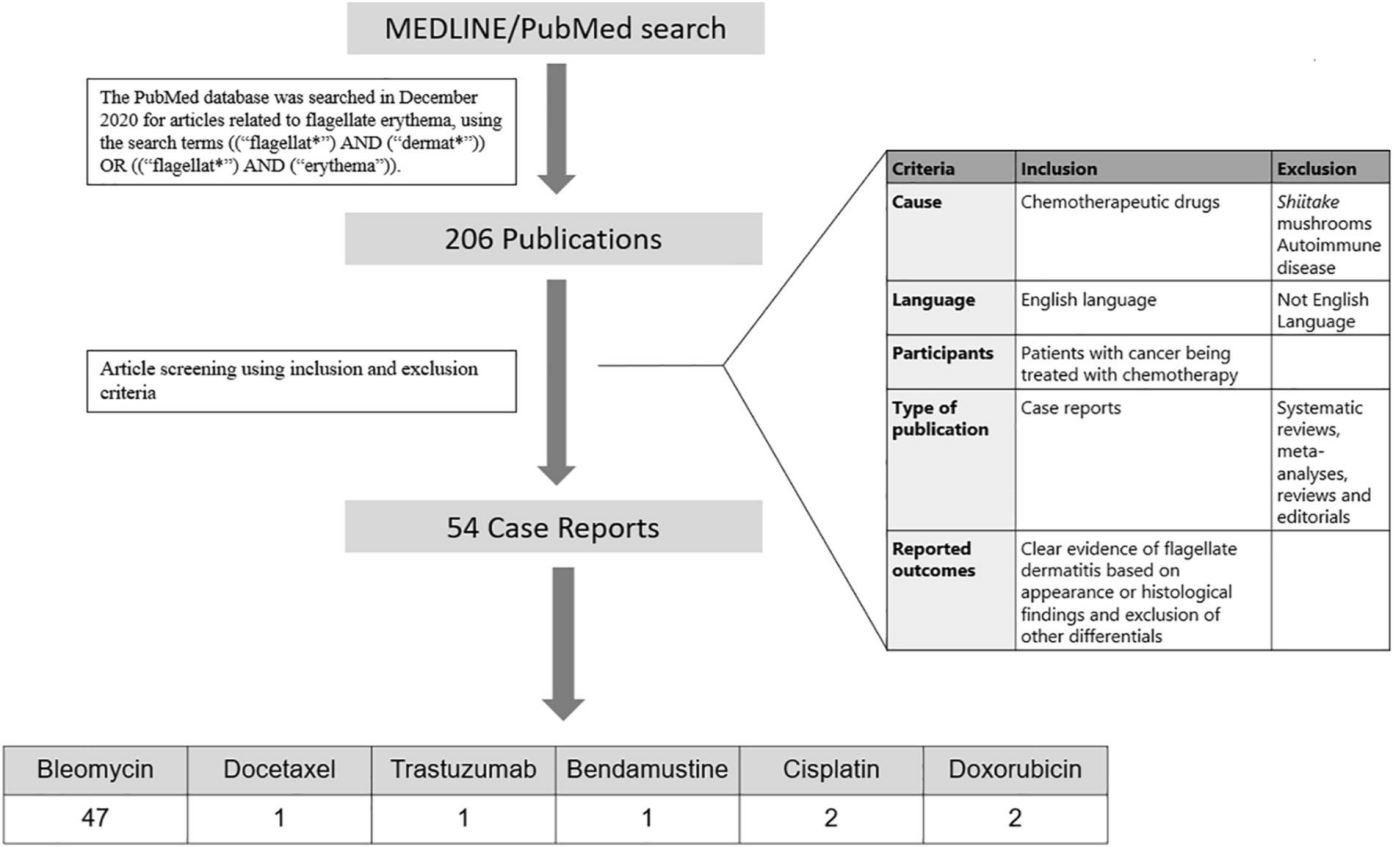
Methodology of literature review. Data from the finalized 54 case reports was collected in Microsoft Excel and statistical analysis was performed using a *Z* score calculator to compare two population proportions, where appropriate

The PubMed database was searched in December 2020 for articles related to flagellate erythema, using the search terms ([‘flagellat*’] AND [‘dermat*’] OR [‘flagellat*’] AND [‘erythema’]). The search yielded 206 publications which were screened by their title and abstract, after which 47 publications describing 54 individual cases fulfilled our inclusion criteria were identified (Figure 1). Data was collected in Microsoft Excel and statistical analysis was performed, using a *Z* score calculator to compare two population proportion.

## 4 RESULTS

### 4.1 Cohort characteristics

**FIGURE 2:**
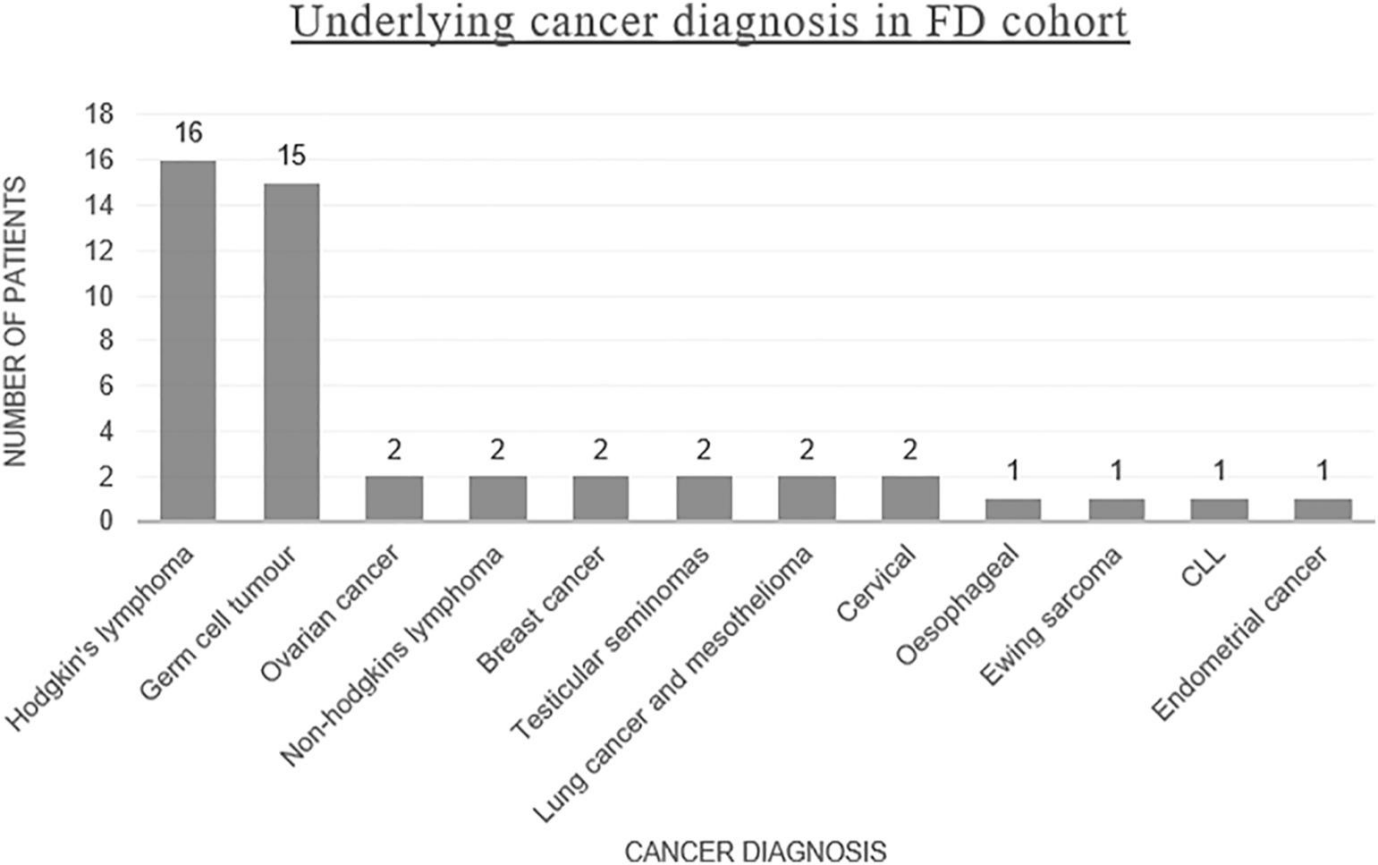
Bar chart summarizing the frequency of underlying cancer diagnosis occurring in our cohort of flagellate dermatitis patients, *n* = 47

The median age of our patients at presentation was 32 years, (range 10–75), with a peak in the 20–39 age group. 54% (29/54) of the cohort were females and 46% (25/54) were males. The two most common underlying cancer diagnoses were Hodgkin's lymphoma (34%) and germ cell tumours (32%) (Figure 2).

### 4.2 Clinical presentation

#### 4.2.1 Body distribution

**FIGURE 3:**
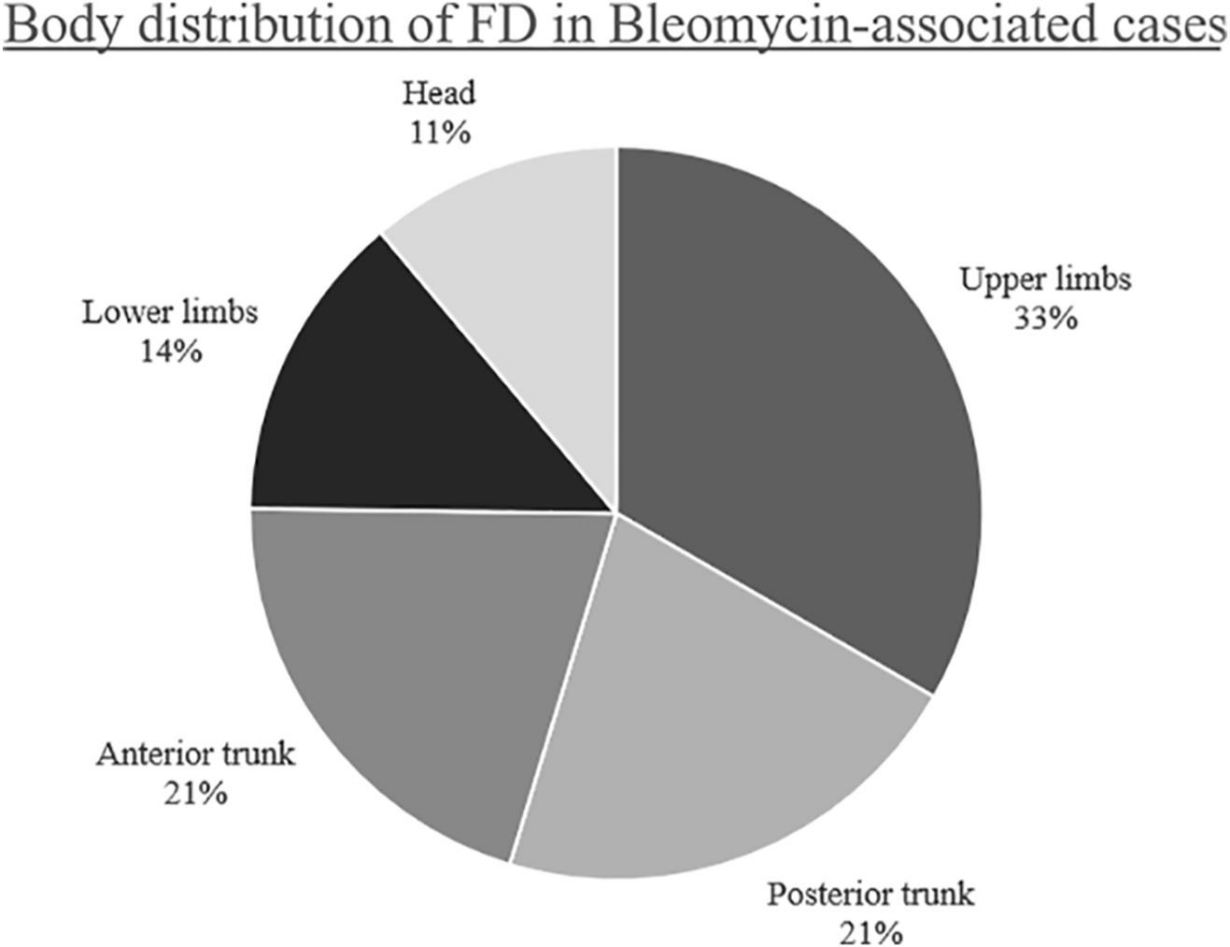
Pie chart demonstrating relative body distribution of flagellate dermatitis in patients treated with bleomycin, *n* = 37

There is no characteristic body distribution.^3^ Most lesions occur on the limbs and the trunk in those patients treated with bleomycin (Figure 3). The distribution in non‐bleomycin patients was also variable, as summarized in Table 2.

**TABLE 2:** Body distribution of flagellate dermatitis in patients treated with chemotherapeutics other than bleomycin

Anti‐neoplastic agent	Number of cases	Location of lesion
Cisplatin	2	Back and shoulders^8^
Docetaxel	1	Back and flank^7^
Trastuzumab	1	Chest, abdomen, and limbs^6^
Doxorubicin	2	Flexural side of the left elbow, the left breast, and the left knee and thigh^4^, ^5^
Bendamustine	1	Central upper and lower back and upper and lower limb extremities^9^

#### 4.2.2 Associated symptoms

Fifty‐one percent (19/37) of bleomycin cases reported pruritus with dermatographia occurring in 14% (5/37) and scalp alopecia in 8% (3/37). In addition, one patient developed horizontal pigmented bands on his nails. Mucosal changes were not noted in any of the 54 cases. In all non‐bleomycin FD cases (*n* = 7), pruritus was present, but dermatographism was not.

#### 4.2.3 Onset of rash

**FIGURE 4:**
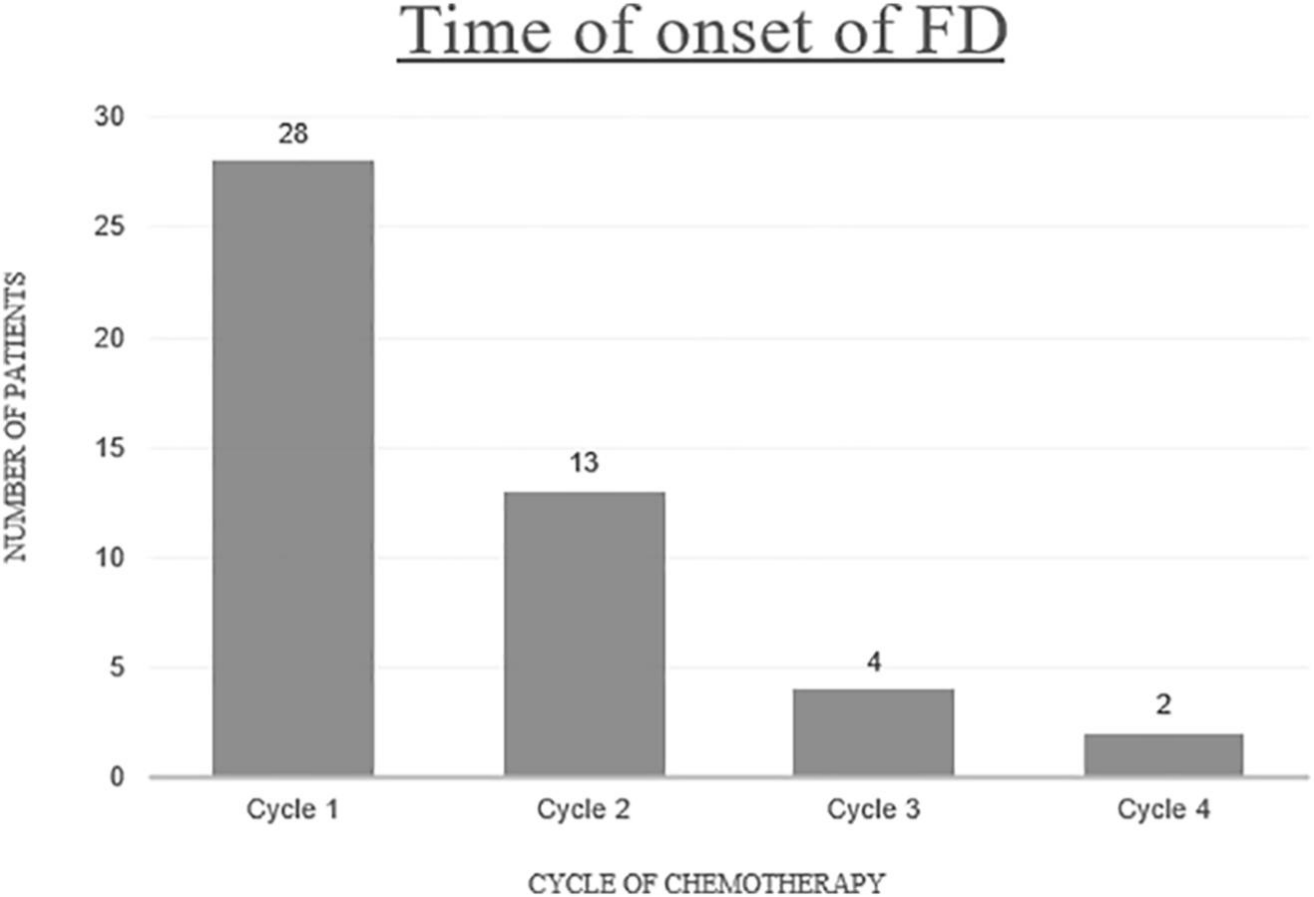
Concentration of events per chemotherapy cycle, *n* = 47

The onset of FD varied from a few hours to up to 6 months following chemotherapy initiation.^8^, ^13^ Interestingly, all who developed FD within 72 h of chemotherapy administration were females (*n* = 7). Most cases of FD occurred after the first cycle of chemotherapy, but some presented as late as the fourth cycle^3^ (Figure 4).

### 4.3 Skin biopsies

A skin biopsy was taken in 41% (22/54) of cases and there was no significant difference between genders (11/29 females vs. 11/25 males, *p* = 0.653). Those who presented within 72 h were not significantly more likely to receive a biopsy (5/7, 71%) compared to those with later onset (17/27, 63%, *p* = 0.674).

### 4.4 Management

**FIGURE 5:**
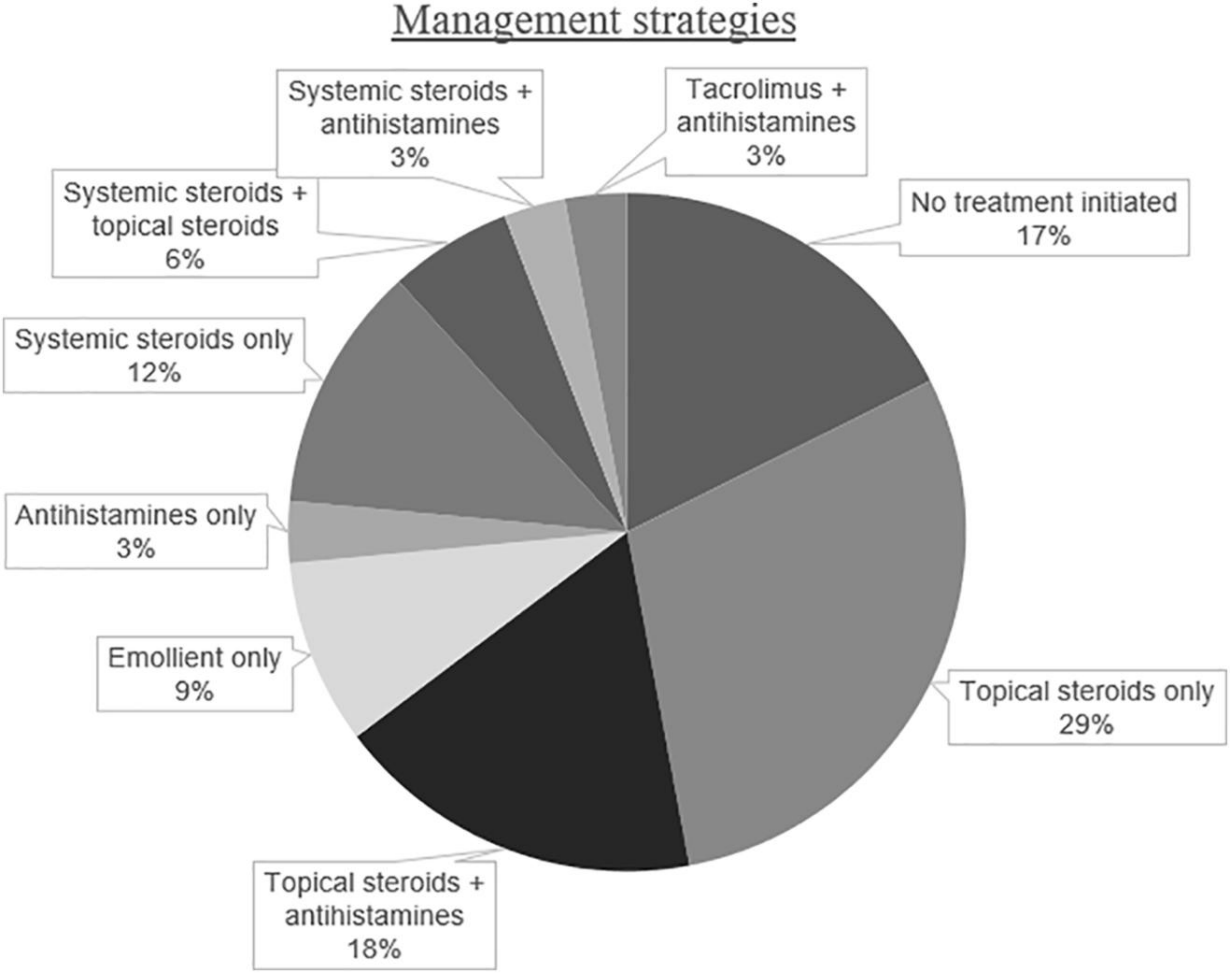
Frequency of different management strategies employed in our cohort *n* = 34. For 20 patients, no specific information on management was given

Most patients received topical steroids (Figure 5). Systemic steroid use was not associated with presentation within 72 h (*p* = 0.365), having a biopsy (*p* = 0.615), or gender (*p* = 0.542). Emollients were not found to be helpful. The length of treatment was dependent on the clinical response.

In most cases, the rash resolved spontaneously within weeks to 4 months following the discontinuation of the causative drug, but post‐inflammatory hyperpigmentation took longer to resolve than acute pruritic erythema. Thirty‐seven cases stated whether chemotherapy was continued or not. Chemotherapy was stopped in 62% (23/37), continued in 32% (12/37) and given as a one‐off preparation in 5% (2/37). The effect of chemotherapy cessation on rash outcomes is variable; in one case, continuance of bleomycin led to recurrence of the original lesions and development of novel lesions.^12^ Alternatively, in a trastuzumab‐induced case, managed by both topical and systemic steroids, the lesions resolved rapidly despite continuance of chemotherapeutics.^6^

## 5 DISCUSSION

Localized cutaneous accumulation of bleomycin may underlie the pathophysiology of bleomycin‐induced FD. Trauma, occurring from pressure sores or scratching, may cause vasodilation leading to localized concentration of chemotherapeutics.^14^ It has been hypothesized that the flagellate pattern may be explained by scratching secondary to pruritus.^3^ However, efforts to provoke FD by irritation of the skin (rubbing) provide conflicting results, with most authors unable to induce the reaction.^15^, ^16^ In addition, several cases described FD in the upper back and other areas of the body which are more difficult to reach, making this hypothesis less likely.^17^, ^22^

**FIGURE 6:**
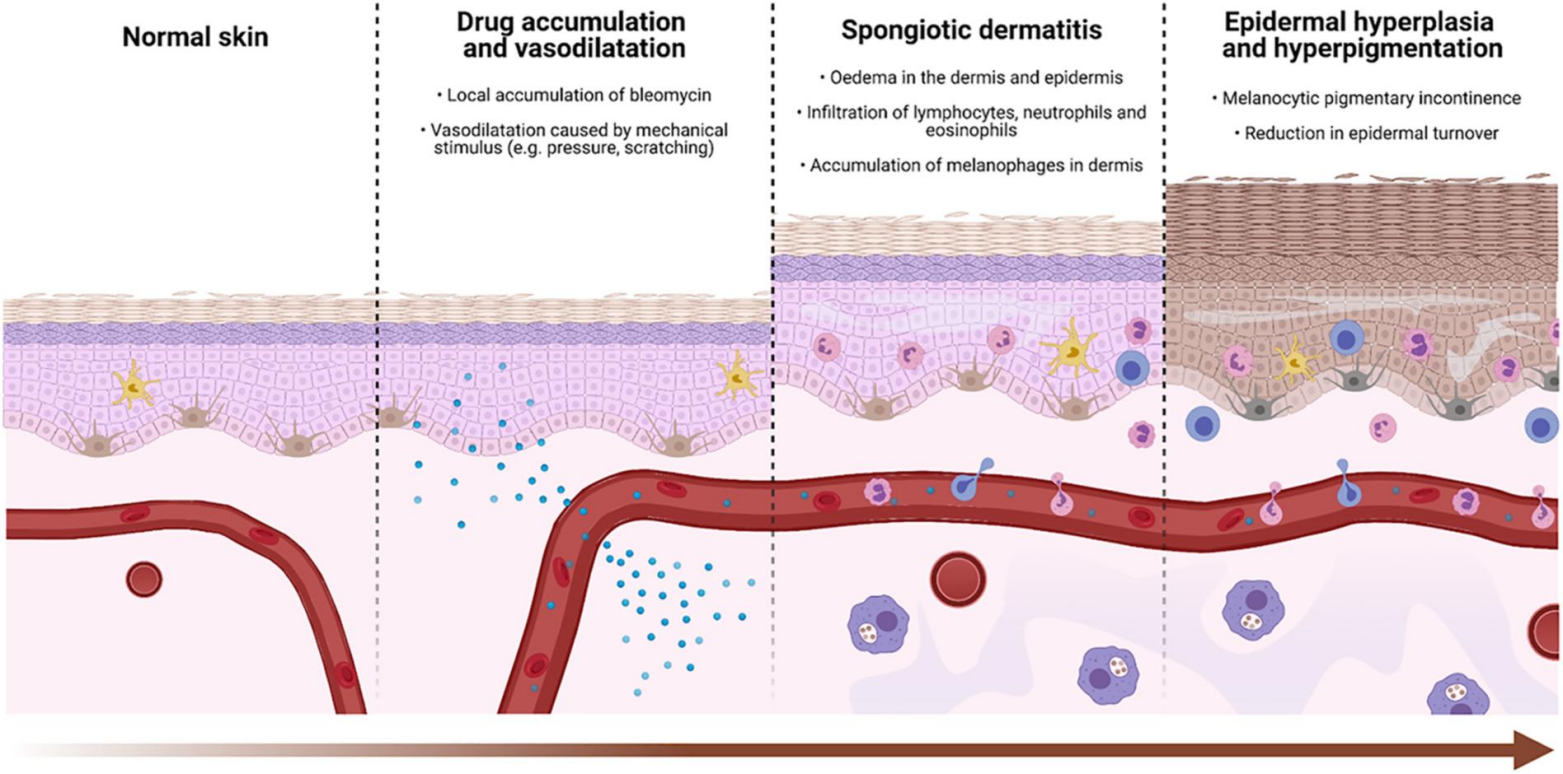
Schematic representation of proposed pathophysiology of bleomycin‐induced flagellate dermatitis

Moreover, bleomycin hydrolase, the enzyme that degrades bleomycin, is expressed only in the epidermis, further enhancing local accumulation.^18^ Histological and ultrastructural studies indicate that bleomycin reduces the epidermal turnover, leading to prolonged contact between melanocytes and keratinocytes.^19^ This may increase melanogenesis and pigmentary incontinence, leading to FD^8^ (Figure 6). The mechanisms through which other anti‐neoplastic agents cause FD are unknown.

The higher prevalence in the 20–39 age group may be accounted by the frequency of germ cell tumours, commonly treated with bleomycin. Hodgkin's lymphoma patients were found to be most affected. This may be explained by the relative deficiency of bleomycin hydrolase reported to occur in Hodgkin's lymphoma.^1^ It has been proposed that reduced levels of bleomycin hydrolase, result in decreased degradation of bleomycin, thereby facilitating antigen presentation and immunogenic reactions.^1^ Additionally, B symptoms like fever and night sweats may act as a trigger for exanthem production.^1^

There is remarkable variability in the time of rash onset. Acute onset may be explained by a toxic cutaneous reaction or a type 1 hypersensitivity reaction involving mast cell degranulation. No skin‐prick tests were attempted in any of the reported cases, but patch testing, which demonstrates a type 4 hypersensitivity reaction, was negative in two cases.^12^, ^20^ It has been hypothesized that FD onset is dose dependant, but most cases developed after the first cycle of chemotherapy. Furthermore, FD has occurred with doses as low as 5IU, making this hypothesis unlikely.^3^

The role of skin biopsies as diagnostic aids in FD is contested. Detailed analysis of the histology of bleomycin‐induced FD has shown variability which depends on the stage of evolution of the lesion and the site of biopsy^1^, ^21^ (Table 3). Due to the non‐specific histology of FD, its characteristic macroscopic appearance and the additional costs and risks to patients, we believe that skin biopsies do not offer a justifiable diagnostic advantage.

**TABLE 3:** Summary of key histological features associated with flagellate dermatitis, modified from Ziemer et al.^1^

Frequency	Features
Common	• Inconspicuous epidermal or spongiotic dermatitis with superficial lymphocytic infiltrate and/or neutrophilic or eosinophilic granulocytes and/or
	• Presence of dermal oedema and/or
	• Presence of melanophages in the papillary dermis and/or
	• Epidermal hyperpigmentation
Occasional	• Presence of necrotic keratinocytes and/or
	• Vacuolar degeneration at the dermal‐epidermal junction
Rare	• Presence of focal acantholysis and/or
	• Presence of leukocytoclasia

Addressing pruritus is the main indication for treatment which may involve topical or systemic corticosteroids and oral antihistamines, sometimes accompanied by a decision to stop chemotherapy. Although cessation of chemotherapy appears to be effective in treating FD, it is worth noting that a delay in chemotherapy by as little as 4 weeks has been associated with increased mortality.^22^ However, in cases where chemotherapy cessation is not acceptable, it appears that a combination of prophylactic topical and systemic steroid treatment may reduce the risk of FD recurrence.^6^ Thus, the decision to stop or continue the chemotherapeutic regime should involve a multidisciplinary discussion and a careful risk–benefit analysis, tailored to the individual patient. Finally, heat exposure should be avoided as it can cause a recurrence.^1^

## 6 CONCLUSION

FD is associated with various chemotherapeutic agents and has a characteristic, whip‐like appearance with variable body distribution. Localized skin accumulation of the drug due to vasodilation and relative deficiency of bleomycin hydrolase predisposes to skin toxicity. Skin biopsies are not essential for diagnosis. Symptomatic management should include topical steroids and oral antihistamines and the patient should be warned about possible long‐term post‐inflammatory hyperpigmentation. Chemotherapy cessation should be determined by a patient‐specific risk–benefit analysis.

## CONFLICT OF INTEREST

The authors declare no conflict of interests.

## AUTHOR CONTRIBUTIONS

**Deeya Kotecha**: Conceptualization; Data curation; Methodology; Visualization; Writing – original draft; Writing – review & editing. **Anastasia Constantinou**: Conceptualization; Data curation; Formal analysis; Methodology; Visualization; Writing ‐ original draft; Writing – review & editing. **Panayiotis Laouris**: Conceptualization; Data curation; Methodology; Visualization; Writing – original draft; Writing – review & editing. **Bruno de Paula**: Conceptualization; Methodology; Supervision; Visualization; Writing – review & editing.

## Data Availability

The data that support the findings of this study are available from the corresponding author upon reasonable request.
